# The Complete Genome Sequence of *Polystichum acrostichoides* (Dryopteridaceae, Polypodiales)

**DOI:** 10.56179/001c.55604

**Published:** 2022-11-12

**Authors:** Sana Raoui, Stacy Pirro, Saaïd Amzazi, Hassan Ghazal

**Affiliations:** 1 Laboratory of Genomics and Bioinformatics, Faculty of Pharmacy, Mohammed VI University of Health Sciences; 2 Laboratory of Human Pathologies Biology, Department of Biology, Mohammed V University; 3 Biodiversity, Iridian Genomes; 4 National Center for Scientific and Technical Research

**Keywords:** fern, genome, assembly

## Abstract

*Polystichum acrostichoides* is a perennial, evergreen fern, commonly found in woodlands, stream banks, and rocky slopes in eastern North America. We present the whole genome sequence of this species. Illumina sequencing was performed on a leaf tissue sample from a single plant collected in Maryland, USA. The reads were assembled using a *de novo* method followed by a finishing step using series of references from related species. The raw and assembled data are publicly available via GenBank: Sequence Read Archive (SRR18053988) and Genome Assembly (JAOYMV000000000).

## Introduction

*Polystichum acrostichoides* is a perennial, evergreen fern native to eastern North America, from Nova Scotia west to Minnesota and south to Florida and eastern Texas. It is one of the most common ferns in eastern North America, being found in moist and shady habitats in woodlands, stream banks and rocky slopes. This species has a tufted, clumping habit, with its fronds arising from a central growth point.

## Methods

Leaf tissue from a single plant collected in Maryland, USA was used for this study. DNA extraction was performed using the Qiagen DNAeasy genomic extraction kit using the standard process. A paired-end sequencing library was constructed using the Illumina TruSeq kit according to the manufacturer’s instructions. The library was sequenced on an Illumina Hi-Seq platform in paired-end, 2 × 150bp format. The resulting fastq files were trimmed of adapter/primer sequences and low-quality regions with Trimmomatic v0.33 (Bolger, Lohse, and Usadel 2014). The trimmed sequence was assembled by SPAdes v2.5 (Bankevich et al. 2012) followed by a finishing step using Zanfona (Kieras, O’Neill, and Pirro 2021).

## Data Availability

The genome assembly yielded a total sequence length of 450,652,285 bp.
